# A Combinatorial Approach to Biophysically Characterise Chemokine-Glycan Binding Affinities for Drug Development

**DOI:** 10.3390/molecules190710618

**Published:** 2014-07-22

**Authors:** Tanja Gerlza, Bianca Hecher, Dalibor Jeremic, Thomas Fuchs, Martha Gschwandtner, Angelika Falsone, Bernd Gesslbauer, Andreas J. Kungl

**Affiliations:** 1 Institute of Pharmaceutical Sciences, Karl-Franzens-University Graz, Humboldtstrasse 46, A-8010 Graz, Austria; 2 ProtAffin Biotechnologie AG, Reininghausstrasse 13a, A-802 Graz, Austria

**Keywords:** chemokines, fluorescence spectroscopy, glycosaminoglycans, heparan sulfate, surface plasmon resonance

## Abstract

Chemokine binding to glycosaminoglycans (GAGs) is recognised to be an important step in inflammation and other pathological disorders like tumor growth and metastasis. Although different ways and strategies to interfere with these interactions are being pursued, no major breakthrough in the development of glycan-targeting drugs has been reported so far. We have engineered CXCL8 towards a dominant-negative form of this chemokine (dnCXCL8) which was shown to be highly active in various inflammatory animal models due to its inability to bind/activate the cognate CXCL8 GPC receptors on neutrophils in combination with its significantly increased GAG-binding affinity [1]. For the development of GAG-targeting chemokine-based biopharmaceuticals, we have established a repertoire of methods which allow the quantification of protein-GAG interactions. Isothermal fluorescence titration (IFT), surface plasmon resonance (SPR), isothermal titration calorimetry (ITC), and a novel ELISA-like competition assay (ELICO) have been used to determine K_d_ and IC_50_ values for CXCL8 and dnCXCL8 interacting with heparin and heparan sulfate (HS), the proto-typical members of the GAG family. Although the different methods gave different absolute affinities for the four protein-ligand pairs, the relative increase in GAG-binding affinity of dnCXCL8 compared to the wild type chemokine was found by all methods. In combination, these biophysical methods allow to discriminate between unspecific and specific protein-GAG interactions.

## 1. Introduction

Composed of repeating disaccharide units with alternating uronic acid- (either d-glucuronic acid or l-iduronic acid) or galactose- and hexamine- (d-glucosamine or d-galactosamine) building blocks, glycosaminoglycans (GAGs) are linear polysaccharides varying in length and molecular weights ranging from 10 to 100 kDa [2,3]. Additional enzymatic post-polymerization reactions (sulfation, desulfation and acetylation reactions) at preferred sites of the disaccharide backbone further increase the structural heterogeneity conferring highly negative charge to defined regions [4]. The main classes of GAGs are heparin and heparan sulfate (HS), chondroitin sulfate (CS), dermatan sulfate (DS), keratan sulfate (KS) and the non-sulfated hyaluronic acid (HA) [5]. Contrary to heparin, which shows an overall high degree of sulfation, and contrary to hyaluronic acid, which is completely unsulfated, heparan sulfate and chondroitin sulfate consist of alternating high sulfated and low sulfated regions. Covalently O- linked to core proteins GAGs form so called proteoglycans which are found on cell surfaces, in the basement membranes of tissues and in the extracellular matrices where they are known to play crucial roles in biological and pathophysiological processes that are important for cell development, growth, regulation of proliferation and differentiation, signalling, mediation of cell adhesion and migration, *etc.* [6,7]. Heparan sulfate and chondroitin sulfate are the major GAG members on cell surfaces and are therefore important interaction partners of chemokines.

Chemokines are a class of small, basic proteins with molecular weights ranging from 8 to 12 kDa [8]. They can be distinguished into inflammatory chemokines that are induced by pro-inflammatory stimuli such as lipopolysaccharide, tumour necrosis factor α (TNF-α), and interleukin 1 (IL-1) or up-regulated by interferon γ (IFN-γ) and homeostatic chemokines. In the course of inflammation, chemokines are released and bind to endothelial cell surface GAGs of blood vessels [9]. Thus chemokine gradients are formed and chemokine-specific leukocytes are attracted to be activated for subsequent extravasation into the surrounding, inflamed tissue [10]. Homeostatic chemokines, in contrast, are constitutively expressed in lymphoid tissue and direct trafficking and homing of lymphocytes and dendritic cells within the immune system.

The binding of chemokines to GAGs is driven mainly by electrostatic forces which lead to high affinity but commonly rather unspecific interactions. Electrostatic interactions occur between basic amino acids and the highly sulfated S-domains of the polysaccharide. Specificity in protein-GAG interactions results from van der Waals and hydrogen bonding forces between the less charged regions of the polysaccharide and respective amino acids of the protein [11]. With a few exceptions (like antithrombin-3 and FGF2, [12,13,14] only very little is known about the exact structure of a human GAG target sequence for a given GAG-binding protein. Great efforts are currently made to overcome limitations in sequencing technologies as well as in preparation protocols for feasible amounts of human GAGs, which would certainly be the next decisive step in understanding the biological and pathological role of these polysaccharides.

Despite the unclear picture regarding specific GAG ligands, chemokine binding to GAGs has been shown to be indispensable for the protein's biological activity, for reasons of localization/ immobilisation within the blood stream, conformational change, and oligomerisation. As chemokines are involved in numerous biological and pathological processes, e.g., inflammation, tumor growth and metastasis, the need for novel chemokine-targeting drugs is high, but so far no major breakthrough has been reported [15,16,17]. We have developed a protein engineering approach which enables us to produce anti- inflammatory decoy chemokines with higher GAG binding affinities compared to their wild-types but with impaired GPC receptor binding and activation [18,19]. Our mutant chemokines exert their biological function by displacing wild type chemokines from the GAG-coated endothelium thereby forming an inert layer which prevents the attraction of leukocytes thus interfering with the extravasation of these cells into the tissue. We have successfully applied our engineering approach to CXCL8 (interleukin-8), CXCL12 (SDF-1a), CCL2 (monocyte attracting chemokine 1), and CCL5 (RANTES) [1,11,20,21,22] Since our engineering approach is based on increasing the GAG-binding affinity of a selected target protein, we have developed a pool of methods for the quantification of chemokine-glycosaminoglycan interactions. Affinities, as expressed in K_d_ or IC_50_ values, are decisive for the selection of mutant candidates which are selected for further drug development.

Taking CXCL8 and its respective mutant form dnCXCL8 as a case study, we describe here the combined use of our methods to get a picture of these protein-GAG interactions under various conditions. We have applied isothermal fluorescence titration (IFT), surface plasmon resonance (SPR), and ELISA-like competition assays (ELICO) to quantitate the interaction of CXCL8 and dnCXCL8 with heparin and heparan sulfate (HS). An integrated analyses of the results gave a very comprehensive picture of the limitations of each method and showed that a single method could in some cases be misleading in the interpretation of protein-GAG interactions and therefore prohibit the development of suitable inhibitors of protein-GAG binding.

## 2. Results and Discussion

**Figure 1:**
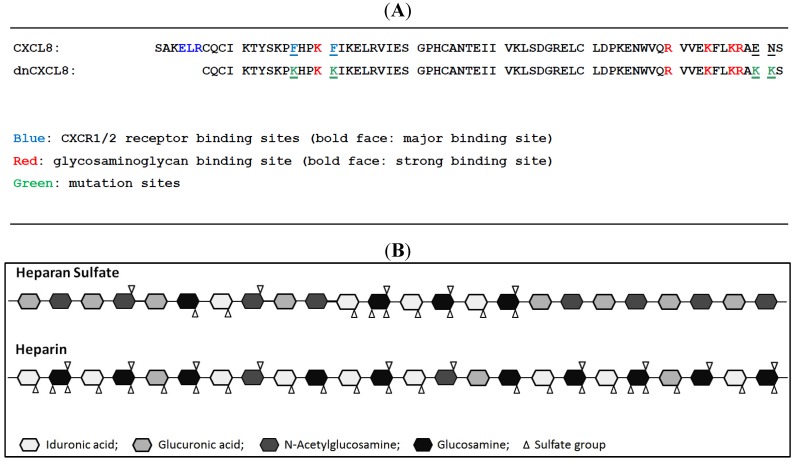
(**A**) Sequence comparison of CXCL8 and dnCXCL8; (**B)** Schematic presentation of heparin and heparan sulfate.

dnCXCL8 is a dominant (= increased GAG binding affinity) negative (= knocked out neutrophil receptor activation) decoy variant of human CXCL8 (see Figure 1). By applying site directed mutagenesis it was engineered to turn pro-inflammatory CXCL8 into an anti-inflammatory chemokine decoy [1] which was developed for the treatment of acute and chronic lung inflammation. Its proposed mode of action is displacing CXCL8 from GAGs on endothelial cells of blood vessels by which the mutant prevents neutrophil extravasation into the surrounding inflamed lung tissue. A high-affinity interaction with GAGs is thus required for dnCXCL8 to be anti-inflammatory. This was therefore characterised here in detail relative to its wild type chemokine counterpart (CXCL8). The sequence of the two proteins is depicted in Figure 1 where we have also displayed the structures of the two GAG ligands under investigation. As can be seen, dnCXCL8 differs from wild type CXCL8 by the higher number of basic amino acids and the lack of the GPCR-binding N-terminal peptide. On the other hand, heparan sulfate (HS) differs from heparin by its overall lower sulfation degree, which leads to an arrangement of high-sulfated domains—responsible for protein binding—and unsulfated domains (other GAG family members such as dermatan sulphate were not considered in this study as we were unable yet to reproducibly biotinylate these molecules which prevents their use in SPR measurements).

### 2.1. Isothermal Fluorescence Titrations

**Figure 2:**
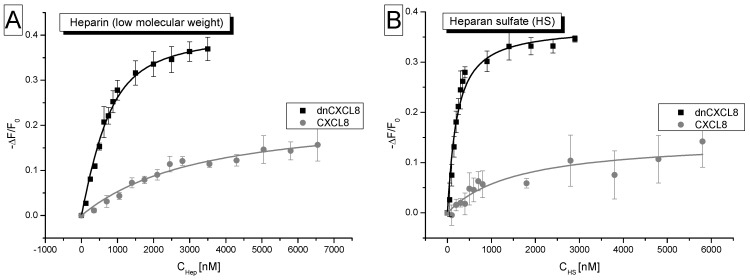
Isothermal fluorescence titration: (**A**) heparin binding isotherms of CXCL8 and dnCXCL8; (**B**) HS binding isotherms of CXCL8 and dnCXCL8 (for K_d_ values see Table 1). On the y-axis, the relative change in fluorescence intensity following ligand addition is displayed: ΔF = F (fluorescence emission at a certain ligand concentration) − F_0_ (fluorescence emission in the absence of ligand).

The intrinsic fluorescence of the single Trp residue located on top of the C-terminal α-helix of CXCL8 was used as sensor signal for GAG-binding. Due to the high fluorescence quantum yield of the tryptophan residue in combination with the environmental sensitivity of the emission, GAG ligand binding can be quantified by this method at very low chemokine concentrations (<1 μM) by a dose-dependent decrease (= quenching) of the fluorescence [23]. The observed quenching is caused by a structural re-arrangement of the chemokine following ligand interaction which impacts on the Trp emission. In Figure 2 the bi-molecular saturation curves of CXCL8 and dnCXCL8 binding to heparin and HS are depicted. For both ligands it is obvious from the different initial slopes in the binding isotherms that dnCXCL8 has a significantly higher affinity to both GAG ligands compared to wild type CXCL8 (see Table 1): 9-fold higher for HS and 8,5-fold higher for heparin. The absolute K_d_ values are in a similar range for dnCXCL8/heparin and dnCXCL8/HS (170 nM and 317 nM, respectively), whereas the values differ quite significantly for the wild type CXCL8/heparin and CXCL8/HS pairs (1,5 μM and 2,7 μM, respectively). This is interpreted by the domination of electrostatic forces in the dnCXCL8 interaction pairs (due to the increased number of basic amino acids in the protein), which are the long-range attraction factors in protein ligand interactions and which are therefore responsible for high affinity binding. Conversely they do not contribute to selective ligand interaction which is mainly attributed to the near-acting hydrogen bond and van der Vaals interactions. Consequently, dnCXCL8 is less discriminative with respect to its ligand compared to the wild type chemokine and therefore binds both, heparin and HS, with similar affinity.

**Table 1:** Isothermal binding and competition values for CXCL8 and dnCXCL8 binding to heparin and to HS.

	Heparan Sulfate (HS)		Heparin	
	K_d_ (nM)	± (SEM)	K_d_ (nM)	± (SEM)
IFT				
dnCXCL8	170	27	317	49
CXCL8	1545	570	2710	600
SPR				
dnCXCL8	255	27	195	14
CXCL8	11500	1050	8305	1700
	IC_50_ (nM)	± (SEM)	IC_50_ (nM)	± (SEM)
ELICO				
dnCXCL8	180	24	130	18
CXCL8	730	59	810	205

Interestingly also the degree of saturation (as expressed in −ΔF/F_0_) is much higher for dnCXCL8 again for both GAG ligands compared to wild type CXCL8. This means that ligand-induced quenching of the Trp residue in dnCXCL8 is much more efficient (approximately 3-fold higher) than for CXCL8. This refers to a significant difference in the structural change induced by GAG binding to the two proteins. We have shown previously by CD-spectroscopy that CXCL8 and dnCXCL8 do not exhibit the same overall three dimensional fold [1]. Therefore, GAG ligand binding is expected to lead to a different structural re-arrangement in the two proteins which impacts in a different manner on the tryptophan emission and thus to a different signal saturation behaviour. The stronger electrostatic attraction between dnCXCL8 and the GAG ligands compared to CXCL8 also contributes to the more efficient ligand-induced fluorescence quenching of the mutant compared to the wild type.

The IFT method is a highly sensitive and robust method to determine protein-GAG binding affinities without chemically or genetically modifying the protein and/or the ligand, but only if the protein under investigation naturally contains a tryptophan residue (if this is not the case, a tryptophan residue can be introduced by genetic engineering or an extrinsic fluorophore like fluorescein can be chemically attached to the protein, e.g., via primary amine chemistry). Depending upon the protein and the mode of interaction, the method is able to discriminate between similar but significantly different polysaccharide ligands like heparin and HS. It is generally easy to implement but cannot easily be automated which would be important for screening purposes (a comparison of the methods described here relating to practical matters is given in Table 2). The stoichiometry of the interaction is not revealed by these experiments, likewise the oligomerisation state of the interacting partners. Both, stoichiometry and oligomerisation, would be important to more deeply characterise the protein-GAG interaction. Especially with respect to the number of binding sites, GAGs are a very special class of bio-macromolecules as they may contain multiple binding sites for a single protein. Because all commercially available GAG preparations consist, in addition, of many different individual molecules which differ in length as well as in overall charge and sulfation patterns, an estimation of binding sites is as impossible as to determine the exact nature of the very specific GAG ligand for a defined protein. However, IFT is a suitable method to characterise protein-GAG interactions for drug development as long as a certain class of GAGs is targeted rather than a well defined GAG oligosaccharide sequence (like in the case of antithrombin III).

**Table 2:** Comparison of the four methods described here relating to practical considerations.

	Amount of Chemokine Needed for One Set of Experiments (*n* = 3)	Sensitivity * (LDL Estimate)	Limitations
**Isothermal Fluorescene titration (IFT)**	50–200 µg	100 nM (chemokine)	intrinsic tryptophan residue which is sensitive to ligand binding (via ligand-induced conformational change); background fluorescence (*i.e.*, due to contaminant proteins); automatisation very difficult
**Surface Plasmon resonance (SPR)**	50–200 µg	100 nM (GAG)	biotinylation of GAGs; GAG chip coating efficiency; better fitting algorithms needed for calculating on and off rates
**ELICO**	20–50 µg (biotinylated chemokine)	200 nM (chemokine)	biotinylation of target chemokine; large amounts of competitor chemokines
**Isothermal titration calorimetry (ITC)**	1 mg	1 µM (chemokine)	large amounts of chemokine; re-buffering of GAG ligand(s) to avoid dilution heat effects

* lower detection limit referring to the lowest concentration of the reporting interaction partner (= chemokine or GAG).

### 2.2. Surface Plasmon Resonance Measurements

**Figure 3:**
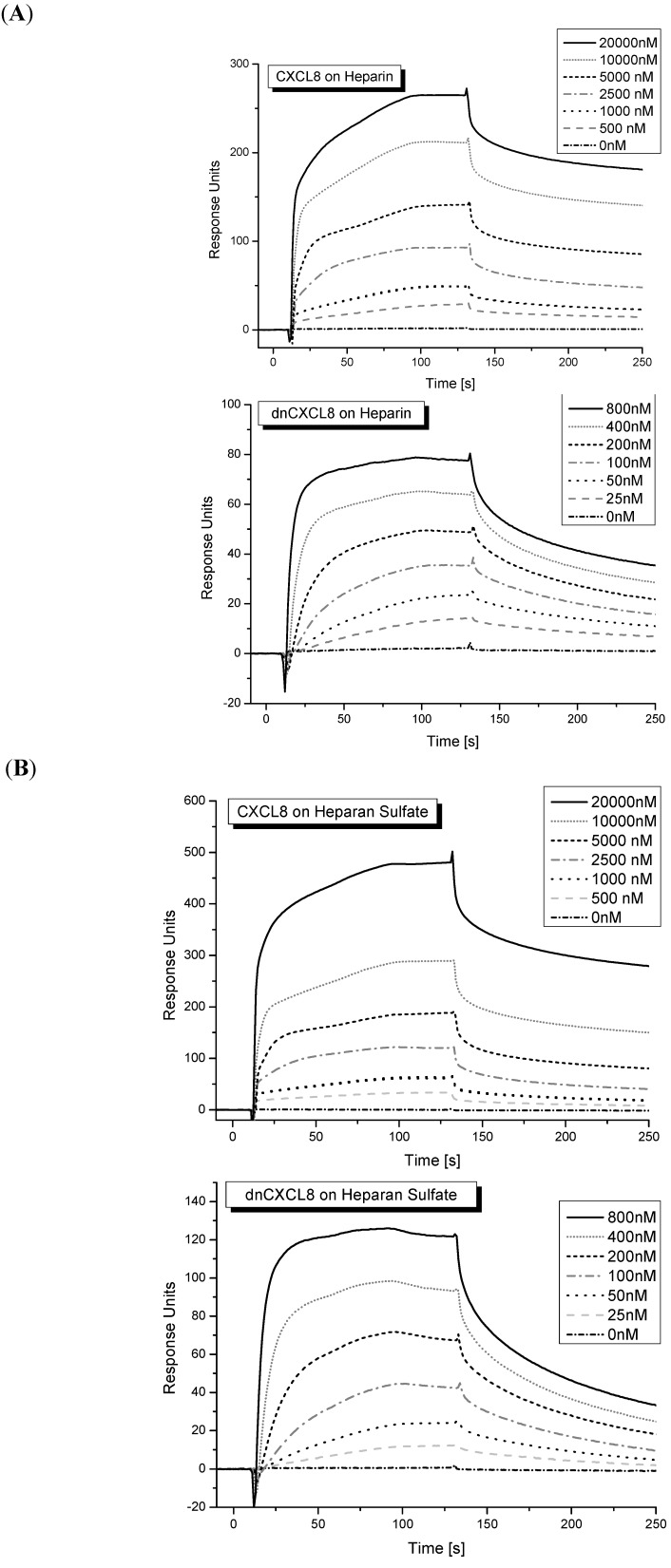
Surface plasmon resonance: (**A**) sensogram of CXCL8 and dnCXCL8 binding to heparin; (**B**) sensogram of CXCL8 and dnCXCL8 binding to HS. The step-wise addition of protein ligands is shown as insert (for K_d_ values see Table 1).

A much more common method in drug development for investigating protein-ligand interactions is surface plasmon resonance (SPR) [24]. One of the major methodological differences compared to IFT is that one of the interaction partners needs to be immobilised. In our case, we biotinylated the two GAG ligands either via their carboxyl groups (HS) or by reductive amination (heparin, see the Experimental Section for further details), depending upon the availability of reducing ends. The biotinylated GAGs were then immobilised on streptavidin-coated SPR chips and then used for chemokine and mutant interaction studies in a typical SPR set-up. This set-up would in principle also allow the determination of kinetic on- and off-rates (kon/koff) on top of equilibrium K_d_ values. In the case of our chemokine-GAG interactions this was not possible due to the lack of a mathematical model which would allow the fitting of rate constants for such interactions (see Figure 3). Since the binding kinetics is most probably influenced by oligomerisation/de-oligomerisation processes of the chemokine (induced by GAG binding) a deconvolution of binding and aggregation processes is too complex to model mathematically.

Typical sensograms obtained for CXCL8 and dnCXCL8 binding to heparin or HS are displayed in Figure 3. A biphasic behavior of the sensograms was observed especially at higher chemokine concentrations which again might be the result of protein oligomer formation that was more dominant at higher chemokine concentrations. The K_d_ values obtained for the four protein-ligand pairs are shown in Table 1. Again, the affinities found for dnCXCL8 to heparin and HS are in a similar range whereas the K_d_ values for CXCL8 binding to these two ligands differ significantly, similar to the results obtained in the IFT measurements (see above). In addition, the relative affinity increase between wild type chemokine and the mutant is much higher for both ligands, *i.e.*, over 40-fold. This much larger difference in K_d_ values between CXCL8 and dnCXCL8 observed in SPR measurements is interpreted as being due to the strong entropic contribution which favours GAG binding only when this otherwise very flexible ligand is immobilised. Interestingly, structurally immobilised GAGs reflect more the situation which is expected *in vivo* where membrane-bound proteoglycans (such as the syndecans and the glypicans) provide GAG “floats” for chemokine binding in order to prevent the molecules from floating around. This quasi-static localisation of chemokines via GAG chains provides the basis for the solid-state chemotactic gradient that is responsible for leukocyte migration *in vivo* [7,18].

The SPR method is a fairly sensitive and very robust method to determine GAG-protein binding affinities. One of the interacting partners, in our case the GAG ligands, need to be chemically modified for the required immobilisation on the SPR chip. This method can, moreover, be automatised which is of great advantage if screening for high-affinity binders is needed. In addition, the kinetics of protein-GAG binding is in principle contained within the data, for which more sophisticated mathematical models need to be developed that take into account the GAG-induced oligomerisation of chemokines.

### 2.3. Isothermal Titration Calorimetry Measurements

**Figure 4:**
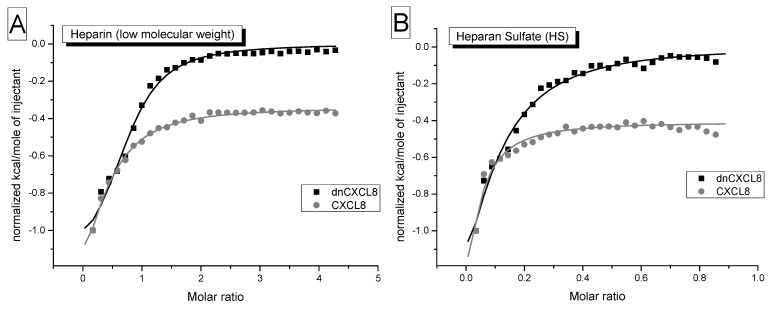
Isothermal titration calorimetry: binding isotherms of dnCXCL8 (black) and CXCL8 (grey) against heparin (**A**) and heparan sulfate (**B**) are shown. It can be clearly seen that the first addition of ligand already generates binding heat which does not allow for a concise data analysis (for further interpretation see text).

Isothermal titration calorimetry (ITC) has been used in the past to determine protein-GAG ineractions [25,26]. In ITC measurements heat is released or absorbed due to ligand binding. Typically solvent and salt displacement as well as conformational changes contribute to binding heats, representing enthalpic and entropic terms, respectively. Due to the low sensitivity of the method, quite high protein concentrations are needed to achieve a sufficiently high signal which is responsive to ligand addition in a concentration-dependent manner. Therefore, a 20-fold higher chemokine concentration was applied in ITC experiments (15 μM) compared to IFT measurements (700 nM). These method-dependent, relatively high protein concentrations induce artificial dimer/oligomer formation which leads to K_d_ values incomparable to IFT and SPR. As can be seen from the binding isotherms obtained by ITC (Figure 4), already the first addition of GAG ligand resulted in a rather large heat release with no baseline formation, which rendered reliable data analysis impossible. Therefore, we were not able to fully explore the potential of ITC experiments by which also the stoichiometry of the interaction (n), the reaction enthalpy (ΔH) and thus entropy changes (ΔS) can in principle be determined. The limitations mentioned above are dependent upon the chemokine under investigation, CCL2 for example exhibits a different oligomerisation behaviour which leads to straightforward analysable ITC data [20,27,28].

### 2.4. ELISA-Like Competition Assays

Since our decoy proteins were designed to displace the corresponding wild type chemokine from its GAG co-receptor, we have developed a novel competition assay which yields IC50 values from displacement curves rather than K_d_ values obtained from bi-molecular binding isotherms. In our assay we have tried to mimic the glycocalix of cell surfaces by coating GAGs (heparin or HS) on specially prepared microtiter plates (Iduron Inc.). We then added biotinylated CXCL8 which was, after washing, displaced either by unmodified CXCL8 or by dnCXCL8 in a concentration-dependent manner. The detection of bound biotinylated CXCL8 at 450 nm is based on a typical ELISA set-up using streptavidin coupled to HRP (see the Experimental Section for further details). For this purpose, we have established a biotinylation protocol that was evaluated using IFT to prove that this labeling did not affect GAG binding (data not shown). In addition, the overall protein fold after biotinylation remained the same as the fluorescence emission spectra did not show any significant wavelength shift (data not shown). We were therefore confident that our labeling procedure yielded structurally and functionally active biotinylated chemokines which still reflect the natural characteristics of the unlabeled protein.

**Figure 5:**
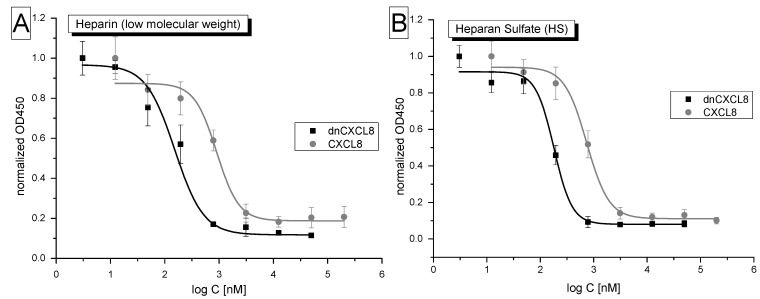
ELICO: Displacement assay of dnCXCL8 (black) in comparison to CXCL8 (grey) competing with biotinylated CXCL8 for binding to heparin (**A**) or HS (**B**).

The displacement curves are summarized in Figure 5. Similarly to IFT and SPR, the relative difference in GAG binding affinities between dnCXCL8 and wild type CXCL8 are reflected also in the competition experiments: dnCXCL8 was a 4-times better competitor for HS, and 6-times better for heparin compared to its wild type CXCL8 counterpart. However, there was no significant difference observed for the two GAG ligands binding to CXCL8. This means that in a competition set-up, both proteins (CXCL8 and dnCXCL8) were equally unable to discriminate between the two GAG ligands. The ELICO assay was originally designed to generate a displacement pattern of one (biotinylated) chemokine against a whole panel of other chemokines and GAG-binding proteins, that is to check for selectivity of protein displacement on the same GAG ligand rather than to discriminate between different GAG ligands. For the purpose of protein discrimination, the ELICO assay was shown to be robust and very sensitive. The major limitation is protein biotinylation which needs to be checked for structure/function conservation by an independent means (such as direct GAG binding monitored by IFT).

## 3. Experimental Section

### 3.1. Materials

GAG binding plates, LMW heparin, HMW heparin, heparan sulfate and dermatan sulfate were purchased from Iduron (Manchester, UK), all chemicals, unless stated otherwise, from Sigma- Aldrich (St. Louis, MO, USA). CXCL8 and DN-CXCL8 (CXCL8Δ6F17KF21KE70KN71K) were produced in house. Phosphate- buffered saline (PBS) pH 7.2 contains 10 mM phosphate buffer and 137 mM NaCl.

### 3.2. Isothermal Fluorescence Titration (IFT)

The titration experiments were performed on a Fluoromax-4 Spectrofluorometer (Horiba, Kyoto, Japan) coupled to an external water bath to ensure constant temperature during the measurements. Protein fluorescence emission spectra were recorded over the range of 300–400 nm upon excitation at 280 nm. The slit widths were set at 5 nm for excitation and emission, scan speed at 500 nm/min and the temperature was set to 20 °C. The use of concentrated GAG oligosaccharide stock solution ensured a dilution of the protein sample less than 5%. Prior to collection of the initial (=unliganded) protein emission spectra, 700 nM protein solutions were prepared from stock solutions and needed to be equilibrated for 30 mins. Following, respective GAG ligands (HS, HMW heparin, DS or LMW heparin) were added in concentrations ranging from 50 nM to 200 nM, the protein solutions were equilibrated for 1 min and fluorescence emission spectra were collected. For background correction the emission spectra of the respective GAG concentrations were collected in PBS buffer only. They were subsequently subtracted from protein emission spectra and the resulting curves were then integrated. The mean values resulting from 3 independent measurements were plotted against the concentration of the added ligand. The resulting binding isotherms were analysed by nonlinear regression using the program Origin (Microcal Inc., Northampton, MA, USA) to the following equation describing a bimolecular association reaction, where Fi is the initial and Fmax is the maximum fluorescence value. K_d_ is the dissociation constant, and [protein] and [ligand] are the total concentrations of the protein and the GAG ligand:

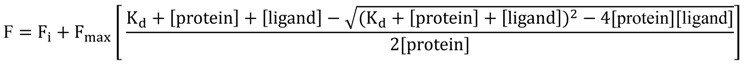



### 3.3. Surface Plasmon Resonance (SPR)

#### 3.3.1. Biotinylation by Reductive Amination for LMW Heparin

LMW heparin (50 mg) was dissolved in 2 M NH_4_Cl (1 mL). CH_3_BNNa (100 mg, Fluka, Buchs, Switzerland) was dissolved in 2 M NH_4_Cl (1 mL) and pipetted into dissolved GAG (1 mL) [29]. The reaction was incubated for 48 h at 70 °C under shaking. Then another portion (50 mg) of freshly weighed out CH_3_BNNA were added to the reaction and incubated for additional 48 h at 70 °C. Dialysis against PBS was performed using Float-a-lyzer (Spectrum Labs, Rancho Dominguez, CA, USA) repeated four times for 16 h. After reducing the volume about 30%–40% using a speed vac concentrator (Eppendorf, Vienna, Austria) the concentration of the GAG was determined by gel electrophoresis as described by Gunay [30]. GAG (50 mg) was coupled to sulfo-NHS-LC-biotin (15 mg, Pierce, Rockford, IL, USA). Depending on the concentration of GAG, the respective molar amount of biotin was weighed out. The biotinylation reaction was performed in PBS (2 mL) on ice for 2 h. To get rid of the unbound biotin the reaction mixture was desalted again in a Float-a-lyzer against PBS for 16 h with four buffer changes and then reduced in volume on a speed vac concentrator. Again the concentration of the biotinylated GAG was determined on a gel as described before [30]. To estimate the quality of biotinylation of different GAG preparations we compared the binding properties with a biotinylated GAG reference batch with known binding properties and quality. The unknown biotinylated GAG was immobilized on the first flow cell under the same conditions than a reference batch on flow cell 2 using a flow rate of 5 µL/min, for a certain contact time, depending on the type of GAG, usually around 60 s. R_max_ and R_immob_ were determined and set relative to the baseline response. Biotinylation efficacy in % was calculated by dividing R_immob_ by R_max_ multiplied with 100. For comparison of batches R_immob_ of the unknown b-GAG was divided by the reference b-GAG that gave a specific immobilization reference value.

#### 3.3.2. Biotionylation of Carboxyl Groups for Heparan Sulfate

Heparan sulfate (10 mg) was dissolved in 0.1 M MES buffer pH 5.0. 1.25 mM Biotin Hydrazid (Pierce) and 6.5 mM EDC were added and the solution was incubated for 2.5 h at RT. Dialysis against PBS was repeated four times for 24 h using a Float-a-lyzer. The quality of biotinylation was determined as described for LMW heparin.

#### 3.3.3. Affinity Measurements

All measurements were performed on a BiacoreX100 system (GE Healthcare, Uppsala, Sweden) at a constant temperature of 25 °C. PBS plus 0.005% Tween (Merck, Darmstadt, Germany) was used as running buffer. C1 sensor chips (GE Healthcare) were pre-treated and washed as described in the manufacturer’s instruction manual. Before immobilizing biotinylated GAGs on the surface of C1 chips, C1 carboxyl groups were activated with EDC/NHS and coated with neutravidin (0.2 mg/mL in acetate buffer pH 4). The remaining active groups were blocked with ethanolamine. Flow runs were set to 5 µL/min and contact time for all reagents to 10 min. b-GAGs were immobilized with a flow rate of 5 µL/min and a concentration of 20 µg/mL. Contact times were varied depending on the used GAG and the determined biotinylation efficacy. The reference cell was only immobilized with neutravidin, serving as control to measure background binding of chemokines. For each binding measurement, 7 different concentrations of the respective chemokine were measured in quadruplicates; the second lowest concentration was injected twice as control. Contact times for all injections and dissociations were 120 s at 30 µL /min over both flow cells. The regeneration solution (1 M NaCl) was enclosed directly after each dissociation time with 30 µL/min and 60 s contact time after each cycle. The data were evaluated using the Biacore Evaluation software 1.0. Affinity constants were assessed by a simple 1:1 equilibrium binding model, where R_eq_ is plotted against the analyte concentration. For fitting the steady state formula that corresponds to the Langmuir adsorption equation was used provided by the Biacore Evaluation Software.

### 3.4. Isothermal Titration Calorimetry (ITC)

The measurements were performed on a VP-ITC Microcalorimeter (MicroCal, Pittsburgh, PA, USA). The protein concentration (CXCL8, dnCXCL8) in the sample cell was 15 µM. Titrants (GAGs) were used in concentrations ranging from 25–360 µM depending on size and assumed number of binding sites. Proteins and GAGs were diluted in PBS buffer and degassed for 10 min at 20 °C in the Thermo Vac (MicroCal) prior to use. The reference cell was filled with degassed double-distilled water. Following parameters were adjusted for the experiments: cell temperature was set to 25 °C; reference power 11.8 µCal/s; initial delay: 60s; stirring speed 270 rpm; feedback mode: high; equilibration options set to fast equilibration and auto. After an initial injection of 2 µL (pre-titration) the following titrating aliquots had a volume of 8 µL with 250 s spacing between each titrant addition to assure signal returning to baseline. The filter period was 2; the duration was 4 s for the pre-titration and 16 s for following titrations. The titrant was added until saturation was observed. The maximum total number of ligand additions was 38.

The integrated heat changes were then plotted against the molar ratio and analyzed with Origin^®^ scientific plotting software version 7.0 (MicroCal) using a One Set of Sites curve fitting model to obtain the association constant (Ka), the stoichiometry (N),the enthalpy (ΔH) as well as the entropy (ΔS) of binding. The dissociation constant (K_d_) and the Gibbs free energy (ΔG) of the binding were calculated using following correlations: K_d_ = 1/Ka, and ΔG = −RT ln Ka.

### 3.5. ELISA-Like Competition (ELICO)

#### 3.5.1. Biotinylation of Chemokines

Prior to chemokine biotinylation a buffer exchange was performed to 0.1 M MES using Amicon Ultracel 3K 4 mL (Millipore, Billerica, MA, USA) to provide optimum reaction conditions. The protein was then incubated with 20 molar excess of EZ Link Penthylamine Biotin (Thermo Scientific, Waltham, MA, USA) and 10 molar excess EDC (Pierce) for 2 h at room temperature and low agitation. Desalting was performed using ZEBA desalting columns (Pierce) according to manufacturer’s protocol. Biotinylation grade was determined using a Biotin Quantification kit (Pierce) and protein concentration was determined by photometric measurements.

#### 3.5.2. ELICO Protocol

GAG (2.5 µg) was diluted in PBS (137 mM NaCl, 10 mM phosphate buffer, pH 7.2) and coated on specially prepared Iduron plates over night at room temperature. After a washing step using an automatic platewasher (Tecan, Männedorf, Switzerland), 250 nM biotinylated CXCL8 was incubated 1 h at room temperature with the pre- coated GAG plates. After another washing step to remove unbound biotinylated CXCL8, it was incubated with different competitor concentrations starting from 50 µM to 3 nM diluted in PBS for 2 h at room temperature. To detect the remaining biotinylated CXCL8 we used an ELISA- like setup. After another washing step we incubated the plates with high sensitivity Streptavidin HRP (Pierce) diluted in 0.2% dry milk that binds to the non-displaced biotinylated CXCL8 on the plate. After another hour incubation at RT and removal of unbound Streptavidin by washing, we analysed the plate by adding the substrate Tetramethylbenzidine (TMB), resulting in a blue colour change. After stopping the reaction with sulphuric acid the absorbance at 450 nm was read in a Beckman Coulter DTX 800 Multimode Detector (Vienna, Austria), with correction at 620 nm. The reference (OD_620_) values were subtracted from the sample values (OD_450_) and the Mean and Standard Deviation of the replicates calculated. Data analysis was performed using specialized statistical software Origin^®^ (Microcal Inc.).

## 4. Concluding Remarks

The chemokine-glycosaminoglycan interaction has been recognized as a key step in various biological and pathological processes involving leukocyte migration and extravasation. Different ways of therapeutically interfering with this particular interaction (low molecular weight glycomimetics or antibody approaches) have been investigated, but a major breakthrough has so far not been reported. One of the major problems of protein-GAG interactions in general is the lack of knowledge about the exact nature of the specific GAG ligand. In addition, it is still under debate whether the interaction of proteins with GAGs is specific or unspecific (*i.e.*, based purely on electrostatic interactions), and that there are perhaps only a few exceptions to this rule (like antithrombin-III). In order to investigate the protein-GAG interaction to a level to differentiate between specific and unspecific interactions, a repertoire of methods is needed. Some of the methods have been compared here. Unlike other, quite frequently used methods like heparin affinity chromatography, the biophysical methods described here reveal true affinity or competition constants (K_d_ and IC_50_ values, respectively). This is especially important if the binding potential of various protein mutants to different GAG molecules should be unravelled to a certain degree of sensitivity in the context of drug development. The ITC method, though giving K_d_ values based on heat release/absorption after ligand binding (*i.e.*, the most direct response to ligand binding), the calorimetric method consumes a quite high protein amount. The IFT technique, on the other hand, needs only a fairly low protein concentration and is thus quite sensitive compared to other methods like NMR and analytical ultracentrifugation, but cannot be compared in this respect with radiolabelling methods which are sensitive down into the picomolar protein range. However, either protein or GAG ligand need to be radiolabelled for this purpose. If therefore in IFT experiments intrinsic fluorophores like the tryptophan residue can be used, labelling of the protein can be avoided and therefore a substantial change of the protein's structure (and thus function) can be avoided. Also the SPR set-up used in our study avoids protein labelling and allows, due to immobilisation of the GAG ligand, a view onto the protein-GAG interaction “from the other side”. This means that while in IFT measurements—like in NMR spectroscopy—the affinities are inferred from protein conformational changes, in SPR measurements with immobilised GAGs the interaction strength is detected via the surface reorganisation of the carbohydrate ligand. If therefore, for example, affinities of a defined protein-ligand pair would significantly differ in IFT and SPR measurements, different factors can be assumed to contribute differently to the interaction depending upon the molecular view point (*i.e.*, from the protein like in IFT or from the GAG like in SPR). The most important factors are considered to be enthalpy *versus* entropy (depending upon the overall charge *versus* flexibility of the sensing interaction partner), desolvation and salt release, ligand-induced conformational changes (and consequently oligomerisation). Only the SPR method is able, in principle, to yield on and off rates for the protein-GAG interaction. However, more sophisticated fitting algorithms need to be put in place. Finally, the ELICO method introduced here allows a screening for specificity in a given protein-GAG interaction which is not easily possible in other competitive set-ups like radioligand displacement assays. As the protein interaction partner can be displaced by either other proteins, as described above, but also by other GAG ligands (data not shown) and, moreover, other GAG ligands can be immobilised to serve for the competition experiments, a deeper insight into specificity becomes possible. This will be the task of future experiments.

If therefore possible, we would recommend a combinatorial approach and to start off investigating a protein-GAG interaction by a method which does not influence the structure of the protein nor of the GAG ligand, and which is still sensitive down to a protein concentration comparable to *in vivo* situations (*i.e.*, in the nanomolar range). To our knowledge, only the IFT method using intrinsic tryptophan residues as fluorescence reporters, falls under this category. This method is, on top, very robust, easy to implement, and gives reproducible results. In a second step, if the GAG ligand can be immobilised onto an SPR chip, this method allows to detect and compare affinities from the carbohydrate molecular viewpoint which could give different results and lead therefore to different conclusions about the mode of protein-ligand interaction. Access to kinetic rate constants is a further asset of this method. Specificity is suggested to be challenged in a third step, *i.e.*, once the affinities have already been established. By variation of the competition conditions as well as by the nature of the competitors, conclusions about the specificity of the given protein-GAG pair can be drawn. Finally, high-resolution structure determination of chemokine-GAG complexes by X-ray crystallography or by NMR spectroscopy are needed to ultimately resolve the structure-function relation of such interactions. This was recently demonstrated by Nordsieck *et al.* [31] who have shown that a C-terminal mutation of CXCL8 led to an additional GAG binding site, which could also explain some findings of our study.
